# The Design and Data Analysis of an Underwater Seismic Wave System

**DOI:** 10.3390/s25134155

**Published:** 2025-07-03

**Authors:** Dawei Xiao, Qin Zhu, Jingzhuo Zhang, Taotao Xie, Qing Ji

**Affiliations:** Naval University of Engineering, Wuhan 430033, China; david_engineer@126.com (D.X.); zhangjingzhuo84@sina.com (J.Z.); xtt54321609@163.com (T.X.); jiumingya886@163.com (Q.J.)

**Keywords:** underwater seismic wave, signal detection, detection system, spectral feature extraction

## Abstract

Ship seismic wave signals represent one of the most critical physical field characteristics of vessels. To achieve the high-precision detection of ship seismic wave field signals in marine environments, an underwater seismic wave signal detection system was designed. The system adopts a three-stage architecture consisting of watertight instrument housing, a communication circuit, and a buoy to realize high-capacity real-time data transmissions. The host computer performs the collaborative optimization of multi-modal hardware architecture and adaptive signal processing algorithms, enabling the detection of ship targets in oceanic environments. Through verification in a water tank and sea trials, the system successfully measured seismic wave signals. An improved ALE-LOFAR (Adaptive Line Enhancer–Low-Frequency Analysis) joint framework, combined with DEMON (Demodulation of Envelope Modulation) demodulation technology, was proposed to conduct the spectral feature analysis of ship seismic wave signals, yielding the low-frequency signal characteristics of vessels. This scheme provides an important method for the covert monitoring of shallow-sea targets, providing early warnings of illegal fishing and ensuring underwater security.

## 1. Introduction

In recent years, the development of underwater seismic wave systems has emerged as a critical research domain for underwater ship signal detection. Characterized by their low-frequency attributes, ship seismic wave signals have become a new type of physical field signal feature for ship signal detection, succeeding traditional underwater physical field signals. The ship seismic wave field refers to a physical field composed of vibration waves generated by ships or the marine environment, which propagate through seawater mediums to the seabed and travel along the seawater–sediment interface or within semi-solid or solid media of the seabed [1].

The low-frequency part of ambient noise sources in the ocean both interact and couple with the seafloor, generating background noise in the seafloor seismic wave field, which severely interferes with seismic wave measurements on the seafloor [2]. Only through precise measurement and effective noise suppression can the kinematic information, physical properties, and environmental parameters of marine targets be accurately obtained [3]. Thus, such technologies can provide crucial technical support for marine resource development, environmental protection, and security.

Developed by the OBS Laboratory of Southern University of Science and Technology, a new broadband ocean bottom geophone for passive-source submarine seismic observation features minimized current-induced noise. This geophone has a compact gimbal for precise leveling, and low power consumption for long-term operation on the seabed [4]. Marra et al. have now discovered that ordinary subsea telecommunications cables can also be used to detect seismic wave signals. Ultra-stable lasers send laser light through active fiber-optic cables to detect minute strain changes associated with the passage of seismic waves. This strategy can transform intercontinental fiber-optic cables into subsea strain sensors, thereby significantly enhancing our ability to record seismic wave signals [5]. Wang Xiaohan et al. from Harbin Engineering University designed a hemispherical OBS (ocean bottom geophone) with a double-cabin structure inside, a metal-nylon double-layer casing outside, consistent density with seabed media, and a positive density gradient. This design not only achieves good coupling effects for the better pickup of weak seabed interface waves but also reduces the impact of waterborne noise [6].

The Laboratory for Fundamental Problems of Petroleum Geophysics and Geophysical Monitoring at the Schmidt Institute of Physics of the Earth, Russian Academy of Sciences, is dedicated to detecting and analyzing overpressure zones in loose seabed sediments using ocean bottom geophones. It has successfully identified overpressure zones based on seismic responses and analyzed temporal variations in pore pressure distribution [7].

Although existing undersea seismometer (OBS) technology [4,5,6,7] has realized high-precision seafloor signal acquisition, it still has three major limitations in shallow-sea ship target monitoring scenarios. These include insufficient real-time monitoring: traditional OBS relies on the later recovery of memory cards to acquire data [4], which are unable to meet the real-time demands of ship dynamic monitoring; weak low-frequency noise suppression: seafloor background noise interferes significantly with a ship’s low-frequency seismic waves (<50 Hz) [2], while existing OBS systems mostly use fixed filters, making it difficult to adaptively suppress time-varying noise; and the high cost of shallow sea deployment: OBSs designed for deep sea [6] have redundant performance in shallow sea deployment, leading to a waste of resources.

For real-time monitoring applications, systems like the Deep-Ocean Assessment and Reporting of Tsunamis (DART) project—developed by the Pacific Marine Environmental Laboratory of the National Oceanic and Atmospheric Administration—use a three-tier architecture [8]. The real-time monitoring system adopts a three-tier architecture, with the core component being a Bottom Pressure Recorder located on the seafloor. It uses an acoustic modem to transmit collected data to a surface buoy, which then relays the information to onshore communication networks via satellite links, enabling end-to-end real-time data transmission. However, the system has significant technical limitations: limited data transmission bandwidths and a high packet loss rate lead to the impairment of the integrity of critical monitoring data [9].

To systematically collect seismic wave signals from subsea vessels, the propagation characteristics of ship seismic wave signals need to be deeply investigated in shallow marine environments, expanding the precise measurement technology system for shallow marine information and balancing economic efficiency and low-power design requirements. The Naval University of Engineering has developed a seismic wave detection system adapted for bottom-mounted platforms. This research team conducted equipment deployment, recovery process verification, and multi-scenario signal acquisition experiments in water tank environments and offshore areas near Qingdao. Through standardized operational procedures, the accuracy of sensor signal reception was validated, and raw ship seismic wave data were successfully obtained, laying a measured data foundation for subsequent multidimensional data analysis.

Subsequently, by reading the time-series data stored on the sensor’s SD card, we proposed an improved joint analysis framework integrating Adaptive Line Enhancer–Low-Frequency Analysis (ALE-LOFAR) with Demodulation of Envelope Modulation (DEMON) technology to perform joint time–frequency domain spectral feature analyses on ship seismic wave signals. Experimental results show that the target ship seismic wave signals exhibit significant low-frequency energy concentration characteristics and dense line spectral structures, which proves the feasibility of long-distance and large-data-volume detection. The first successful application of molecular electronic sensors in shallow sea scenarios, balancing low-frequency response and shock resistance, filled in a gap in underwater validation. The modular watertight chamber supports flexible deployment in water depths of 10–100 m at 40% of the cost of a deep-sea OBS.

This study mainly considers two major problems in the real-time transmission of large quantities of data and weak signal extraction in seismic wave monitoring on shallow ships. This research achievement provides critical technical support for constructing early-warning monitoring models in ship seismic wave fields and holds significant scientific and engineering implications for the covert monitoring of shallow-sea targets, providing an early warning of illegal fishing activities and optimizing underwater security systems.

## 2. The Overall Design of the Seismic Wave Detection System

**Figure 1:**
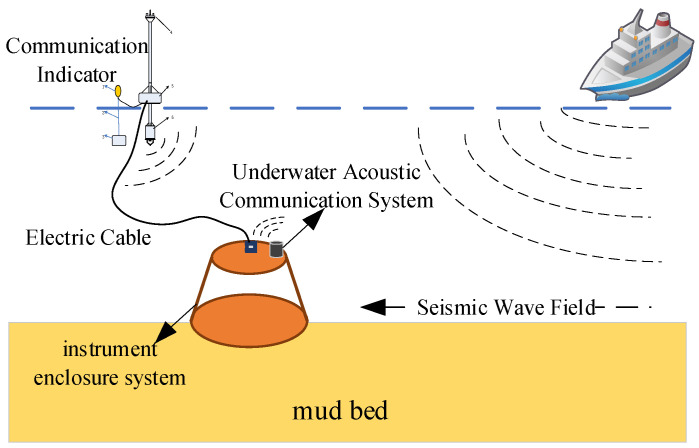
Seismic wave measurement system composition diagram.

The ship seismic wave measurement system employs a surface–underwater collaborative architecture, primarily composed of two components: the surface communication unit and the underwater acquisition unit. The composition of the measurement system is illustrated in Figure 1. The top of the instrument enclosure is equipped with an underwater acoustic communicator, which forms an underwater data transmission link with the acoustic communicator deployed by the data radio communication buoy, enabling the upload of collected data and the download of commands. The surface communication unit is a data radio communication buoy, serving as the surface relay node of the system. Through communication with the radio, it can obtain the operational status of the signal acquisition system and attitude information to determine whether the watertight instrument enclosure has settled on the seabed and stopped shaking. The underwater acquisition unit is the watertight instrument enclosure, acting as the core data acquisition and processing module of the system.

The watertight instrument enclosure adopts a modular design, with its bottom part integrating multiple functional modules required for seismic wave signal acquisition: a high-sensitivity geophone for capturing underwater seismic wave signals; a data acquisition and storage module for analog-to-digital conversion and data buffering; and a 12 V battery providing stable power supply, enabling more than 10 days of underwater acquisition. Lead weights can be added to the bottom of the instrument enclosure according to the deployment depth to facilitate anchoring on the seabed. The watertight instrument enclosure and the data radio are connected by cables and ropes, which not only facilitate data transmission but also prevent the data radio from being carried away by surface currents, avoiding communication interruptions with the bottom module and facilitating recovery [10].

As the surface relay node of the system, the data radio communication buoy establishes a two-way communication link with the remote monitoring platform via a wireless data radio, forming a complete “underwater–surface–remote” three-level data transmission architecture. This hierarchical communication design not only ensures the reliability of data transmission but also, compared with ordinary seismic wave measurement systems, enables the remote monitoring and real-time control of underwater equipment, guaranteeing the long-term stable operation of the system. The remote monitoring platform applies an improved ALE-LOFAR joint framework combined with DEMON demodulation processing to the obtained data, which enhances the visibility of low-frequency signals and facilitates the observation and analysis of shallow-sea ship seismic wave signals [4]. The composition diagram of the seismic wave measurement system design is shown in Figure 1.

Compared to the DART system dedicated to open-ocean tsunami monitoring and systems designed for monitoring nearshore oil/gas facilities, the underwater seismic wave measurement system developed in this study offers two flexible data transmission and connection schemes:

1. It employs the parallel deployment of a cable and a rope to connect the surface data transmission buoy with the subsea watertight instrument enclosure. The cable handles real-time data transmission, while the rope assumes the primary mechanical load. During equipment retrieval, this configuration establishes a redundant mechanical load path, effectively preventing fracture risks caused by single-cable stress concentrations and significantly enhancing system retrievability.
2. It requires only a rope to anchor and connect the surface data transmission buoy to the subsea watertight instrument enclosure. An underwater acoustic modem enables subsea positioning, status confirmation, and controlled separation via designated release mechanisms.

The system’s core advantage lies in its ability to operate both modes concurrently. This dual-mode operation not only ensures data integrity but also achieves high-capacity real-time data transmission from the seabed to the surface.

**Figure 2:**
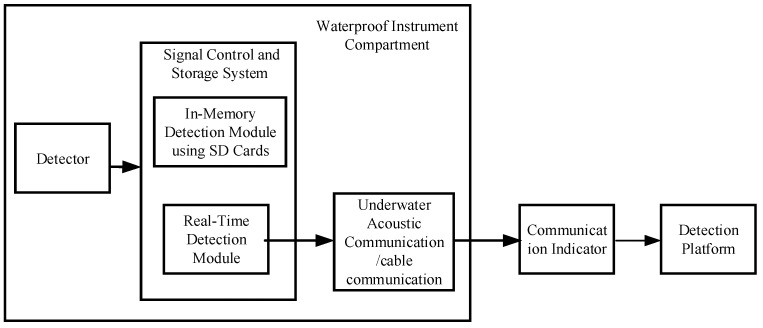
Diagram of underwater seismic wave field monitoring system.

Figure 2 illustrates the operational schematic of the underwater seismic wave field monitoring system. Upon receiving underwater seismic wave signals, the high-sensitivity geophone splits the signal into two paths. One path is stored internally using an SD card, while the other is transmitted via the underwater acoustic communicator, communication cable, surface data radio communication buoy, and finally, to the monitoring platform. This establishes a “subsea-to-surface-to-remote” architecture enabling high-capacity real-time data transmission. The monitoring platform denoises the received data and analyzes the seismic wave field characteristics of target vessels [11].

### 2.1. The Watertight Instrument Enclosure

The watertight instrument enclosure employs a precision engineering structural design, the primary function of which is to provide a reliable operational environment for critical components such as geophones, communication interface modules, underwater acoustic communicators, and seismic signal acquisition and storage circuits while integrating a power management system to enable continuous energy supply. In structural design, engineering factors, including post-settlement stability, the pressure resistance of the cabin, the feasibility of manufacturing processes, and space utilization, were comprehensively considered [12]. A frustoconical cylinder structure was adopted as the main configuration, which is a design that not only optimizes pressure distribution characteristics but also enhances the structural stability to prevent cabin sway caused by water currents, thus minimizing interference. The sealing system utilizes a dual radial O-ring sealing scheme for end caps, effectively ensuring the cabin’s watertight performance [13]. An acoustic release device can be installed at the bottom to facilitate recovery.

Additionally, multifunctional through-holes are pre-installed in the end cap design for the integrated installation of key components such as acoustic communicators and short-circuit switches, enabling reliable data transmission with the underwater acoustic communicator deployed by the data radio communication buoy. Pre-installed cable connection ports also enable optional cabling arrangements with the data radio communication buoy, ensuring that the radio receives comprehensive seismic wave information. A combined Wi-Fi/GPS antenna port is mounted at the top of the housing to facilitate GPS timing and the parameter pre-configuration of sensors before their deployment, guaranteeing normal operation after submersion.

**Figure 3:**
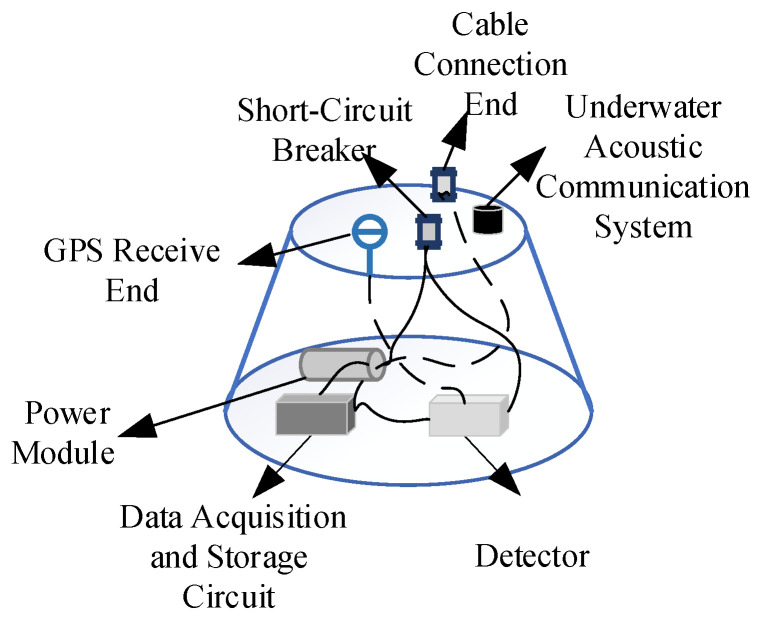
The structure composition diagram of the watertight instrument enclosure module.

Due to the superior detection performance of ship seismic wave signals in shallow seas, the corresponding seismic wave instruments are generally deployed in shallow water areas along beach shores. In terms of dimensions, the instrument features a bottom diameter of 600 mm, a top diameter of 420 mm, a height of 360 mm, and a weight of 100 kg. It can typically be deployed in water depths of up to 32 m, with corresponding counterweights installed at the instrument’s base to adapt to different water depth requirements. The structure composition diagram of the watertight instrument enclosure module is shown in Figure 3. With the addition of a counterweight module, the system can be extended to operate in water depths of up to 100 m, and the pressure chamber was hydrostatically tested to 50 MPa.

In terms of material selection, based on the reliability requirements for marine engineering materials [14] and considering that 316 L stainless steel exhibits excellent corrosion resistance and mechanical strength while having a broad application foundation in marine instrumentation, the core structural components of the watertight instrument enclosure—including the pressure-resistant housing, end caps, and protection frames—are all manufactured from stainless steel. This material selection not only ensures the long-term stable operation of the instrument enclosure in complex marine environments but also meets the pressure resistance and anti-corrosion requirements of deep-sea conditions [15]. The edge chamfer design (R = 20 mm) combined with the surface nano-coating reduces the eddy current noise to below the background noise level (<0.1 μT).

### 2.2. Geophones

A geophone is a sensor that transduces ground vibrations into electrical signals or a mechanical device that converts mechanical oscillations into electrical signals. It produces an electrical analog of the vertical component of ground motion with maximum fidelity. Each geophone consists of a mechanical component and an associated electromechanical transducer with an electrical load. Its amplitude-frequency response must be linear within the meaningful frequency range, and its phase response should also be linear. According to different working principles, geophones can be classified into electromagnetic induction geophones [16], piezoelectric geophones [17], digital MEMSs (Micro-Electro-Mechanical Systems) geophones [18], fiber-optic geophones [19], and molecular electronic sensors [20], among others.

Among these, electromagnetic induction geophones primarily utilize the principle of electromagnetic induction: when the sensor’s housing or metal core undergoes displacement in response to vibration signals, it induces changes in the magnetic field, generating an induced electromotive force and current [21]. Such sensors are vulnerable to external electromagnetic interference, generally have large volumes, and exhibit poor shock resistance. Piezoelectric geophones, by contrast, employ the polarization or resistance change effect generated when a medium is subjected to pressure, converting vibration signals into electrical signal outputs. Their primary advantages include a high piezoelectric coefficient, small size, lightweight, and good shock resistance, though they require integration with a leveler for operation [22].

Fiber-optic geophones offer additional benefits, such as high sensitivity, strong electromagnetic interference resistance, tolerance to high temperatures and pressures, no electric leakage, and easy multiplexing, enabling permanent and real-time online measurements [19]. However, cost and size pose significant barriers to the widespread adoption of fiber-optic sensing technology. MEMS (Micro-Electro-Mechanical System) geophones measure acceleration by detecting mass blocks or vibration masses, typically suspending a sensitive mass block from a reference support via an elastic element [23]. Acceleration acts on the sensitive mass block, with values derived by measuring the displacement of the sensitive mass block, the force exerted by the mass block on the frame, and the force required to maintain its position.

**Figure 4:**
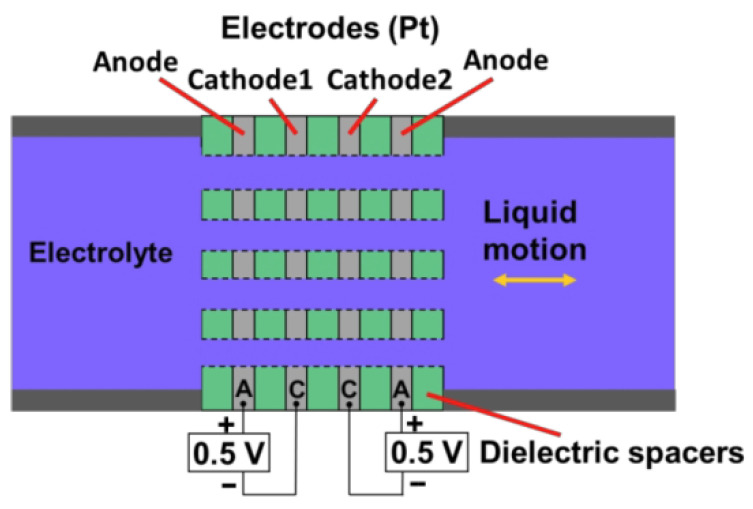
A schematic diagram of the basic molecular electronic sensor element.

The molecular electronic sensor consists of two pairs of anode–cathode electrochemical cells. In an electrolyte-filled channel, there are four anode–cathode–cathode–anode (ACCA) electrodes separated by dielectric layers. Ions in the electrolyte pass through small pores in the electrodes to transfer their charge between the anode and cathode. The difference in cathode currents is used as the output signal of the molecular electronic sensor [24]. A schematic diagram of the basic molecular electronic sensor element is shown in Figure 4.

Molecular electronic sensors enable small-size, low-cost manufacturing, low power consumption, and high shock resistance, achieving low self-noise in low and ultra-low-frequency DC ranges [25]. Meanwhile, the adoption of MEMS microfabrication technology reduces their internal dimensions to as small as 1 micron, thereby enhancing device sensitivity and enabling adaptability to diverse scenarios.

Considering the above types of detectors, the former has high sensitivity, but its cost and power consumption are high. In contrast, the molecular electronic sensor has low cost and low power consumption, which can meet the experimental demand for sensitivity. Therefore, molecular electronic sensors were ultimately selected as the seismic wave measurement device [5].

To effectively collect seismic wave signals from ship targets, it is essential to account for their significant low-frequency characteristics and weak amplitude. Therefore, in designing the seismic wave acquisition system, the system must exhibit excellent low-frequency response performance to ensure the accurate capture of low-frequency components in target signals [26]. Meanwhile, as the vibration signals received by seismic wave sensors have extremely small amplitudes and broad frequency spectra, preprocessing steps (such as amplification and filtering circuits) are critical for enhancing signal quality. Additionally, after analog signals are converted to digital form by an analog-to-digital converter (ADC), precise control and data storage via a microcontroller are required to ensure the stability and reliability of the acquisition process [27].

This study employs a three-channel, 24-bit submarine digital autonomous seismic measurement device. Suitable for underwater seismic and geophysical exploration, this device features a large dynamic range, low self-noise, and energy-saving performance. The sensor’s non-directional characteristics enable convenient deployment in complex topographical environments. The device supports the simultaneous acquisition of three-channel seismic wave signals and is equipped with a positioning and timing device, providing a reliable guarantee for data recording and rapid extraction.

**Figure 5:**
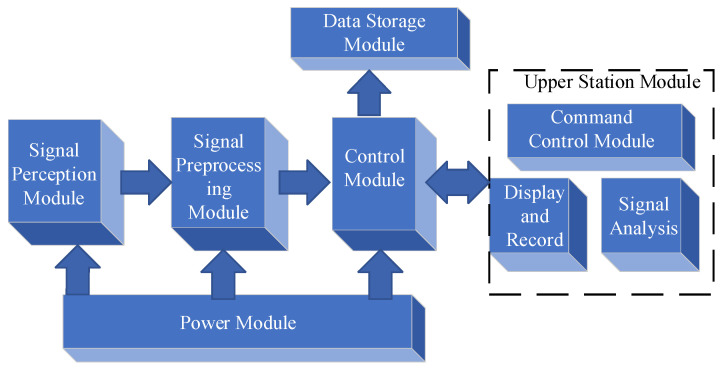
Block diagram of the seismic detector system composition.

A seismic geophone typically consists of multiple modules (Figure 5): a signal sensing module, a signal preprocessing module, a control module, a power supply module, and a host computer module [28]. The signal sensing module primarily includes a seismic wave sensor, an attitude sensor, and an auxiliary output circuit, which are responsible for receiving seismic wave information and performing preliminary processing. The signal preprocessing module comprises an amplification and filtering circuit and a preprocessing circuit for the analog-to-digital converter, which amplifies and filters the acquired analog signals to enhance the signal-to-noise ratio.

The control module generally includes a single-chip microcontroller control center and a debugging auxiliary circuit. The STM32 single-chip microcontroller, manufactured by STMicroelectronics, is selected as the control component, meeting the requirements for high-performance microprocessing and low power consumption, and is used to implement real-time system control and fault diagnosis functions. The data storage module completes the task of storing acquired signals through the output interface of the single-chip microcontroller. The power supply module is powered by a 7.2 V lithium battery (customized by other battery companies in China) and a reference voltage source, providing stable power support for the system [29].

Sensor parameters are shown in Table 1. The seismic geophone of this device can adapt to uneven seabed topographies, maintain stable operation under extreme temperature conditions, and effectively measure seismic wave signals within a frequency range of 1 Hz to 300 Hz [5]. Its measurement performance remains unaffected even when buried. After weak vibration signals received by the sensor undergo preprocessing via amplification and filtering, an AD converter converts the analog signals into digital form. Following signal acquisition and preprocessing, the control system must temporarily store data locally, requiring the system to have data storage capabilities. The storage chip must support the long-term continuous monitoring of the target area via the node; considering the sampling rate and number of channels of the designed system, sufficient storage space is required. Accordingly, this study designs a large-capacity data storage circuit using a MicroSD memory card, which packs and stores data through the storage controller in the microcontroller to achieve long-term and accurate signal storage.

**Figure 6:**
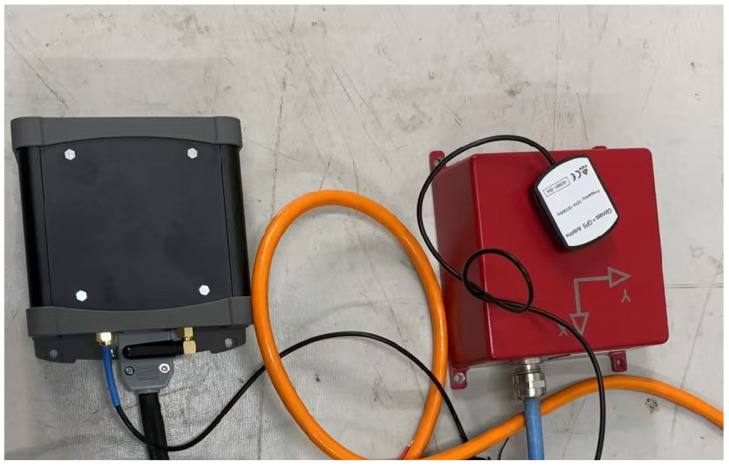
The physical diagram of the detector and data storage system.

The data acquisition system employs the AD7190 chip (manufactured by Analog Devices, Inc., a U.S.-based company) to achieve three-channel synchronous sampling, with the sampling rate flexibly configurable between 125 Hz and 1000 Hz. It features 24-bit resolution and 21-bit effective number of bits (ENOBs) to enable high-precision, low-noise signal quantization. The timing subsystem integrates a multi-mode GNSS receiver (GPS/GLONASS, procured by Party B) with a timing synchronization accuracy of better than 1 microsecond, ensuring the reliability of the time reference. Operating in the continuous acquisition mode, the system supports multiple data storage formats and incorporates 32 GB of non-volatile memory to meet long-term monitoring requirements. Figure 6 shows the physical diagram of the detector and data storage system.

In terms of environmental adaptability, the device can operate stably within a wide temperature range of −40 °C to 55 °C. It is equipped with a three-axis MEMS accelerometer and a three-axis magnetometer for real-time orientation calibration, while humidity, pressure, and temperature sensors continuously monitor the internal safety status of the device to address the challenges of complex underwater environments. By balancing high-precision data acquisition and robustness, this system provides a hardware foundation for long-term autonomous marine monitoring.

Before the seismic wave acquisition system is operational, it requires time synchronization and the initial configuration of the device via a host computer. The working modes are divided into real-time acquisition and timed acquisition, both of which can be effectively controlled by operators through the host computer’s interface. Upon the completion of initialization, the microcontroller begins to operate, and the system initiates autonomous data acquisition processes: the seismic wave sensor first acquires signals, which are amplified by a preamplification circuit and then transmitted to an AD converter for the analog-to-digital conversion of the signals. Subsequently, digital signals are sent to the control chip for sampling, packaging, and local storage. The host computer performs subsequent processing and analysis of target signals by reading the stored data.

After completing data acquisition, the node automatically enters a low-power sleep mode, and the data storage chip also ceases operation, marking the conclusion of the target monitoring process. Target signals can be retrieved by reading data from the memory card via the host computer.

### 2.3. Underwater Acoustic Communicator

To achieve underwater acoustic communication in a specific sea area, the underwater acoustic communicator must cover the depth of the sea area while comprehensively considering factors such as the working bandwidth, modulation scheme, and transmission attenuation [30]. This design of the underwater acoustic communicator selects a working frequency band of 9 kHz to 15 kHz, with a maximum communication distance of 10 km and adaptive communication rates ranging from 320 bps to 6 kbps (supporting programmable modulation via MFSK, single-carrier QPSK, and OFDM). Pressure-resistant housing has dimensions of <100 mm × ∅60 mm, an operating depth >200 m, transmitting/receiving sampling rates of 300 kSPS and 350 kSPS, respectively, a built-in 128 GB solid-state drive (supporting continuous storage >500 h), a maximum transmit power >150 W, and standby power consumption <4 mW, thus meeting the requirements for long-distance and long-duration communication.

**Figure 7:**
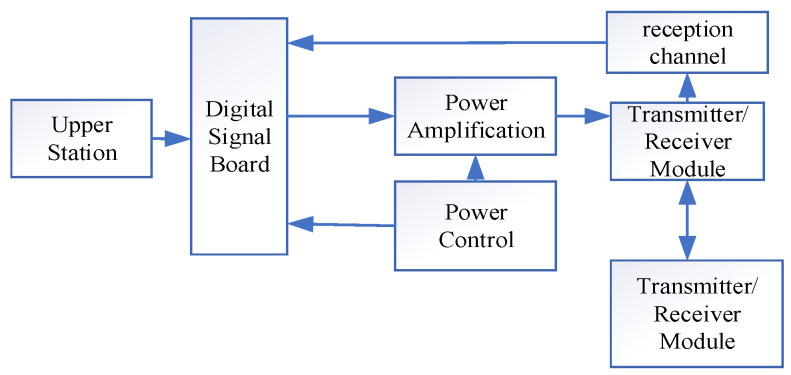
Block diagram of the underwater acoustic modem electronic system.

The electronic system design block diagram of the underwater acoustic communicator is shown in Figure 7. The electronic system design of the underwater acoustic communicator consists of modules such as a digital signal board, power amplifier circuit, and power control.

Through subsequent sea trials, the device achieved reliable communication at 6 kbps within a distance of 10 km. Its compact pressure-resistant structure and low power consumption characteristics meet the deployment requirements of underwater mobile platforms. The open software architecture supports user-defined communication protocols, providing an experimental foundation for algorithm optimization in complex marine environments.

### 2.4. Data Transmission Radio Communication Buoy

The buoy system is a set of wireless communication watertight buoy devices. It adopts an aluminum alloy watertight cabin with a built-in wireless communication module and power module. Dedicated watertight connectors are installed externally. It can be regarded as a relay buoy, which transmits data from underwater equipment through the wireless communication module, realizes real-time synchronization and positioning via GPS, and uses wireless communication for monitoring and controlling the status of the measurement system, thereby achieving remote control [31].

The main functions of the buoy system are as follows:

1. It is equipped with a built-in data transmission radio (frequency: 223–235 MHz) to enable wireless communication capabilities;
2. It incorporates a large-capacity rechargeable lithium battery pack (22.2V@32Ah), which can meet the long-term operational requirements of users;
3. It is equipped with a mechanical watertight switch, enabling the convenient on/off control of the equipment.

**Figure 8:**
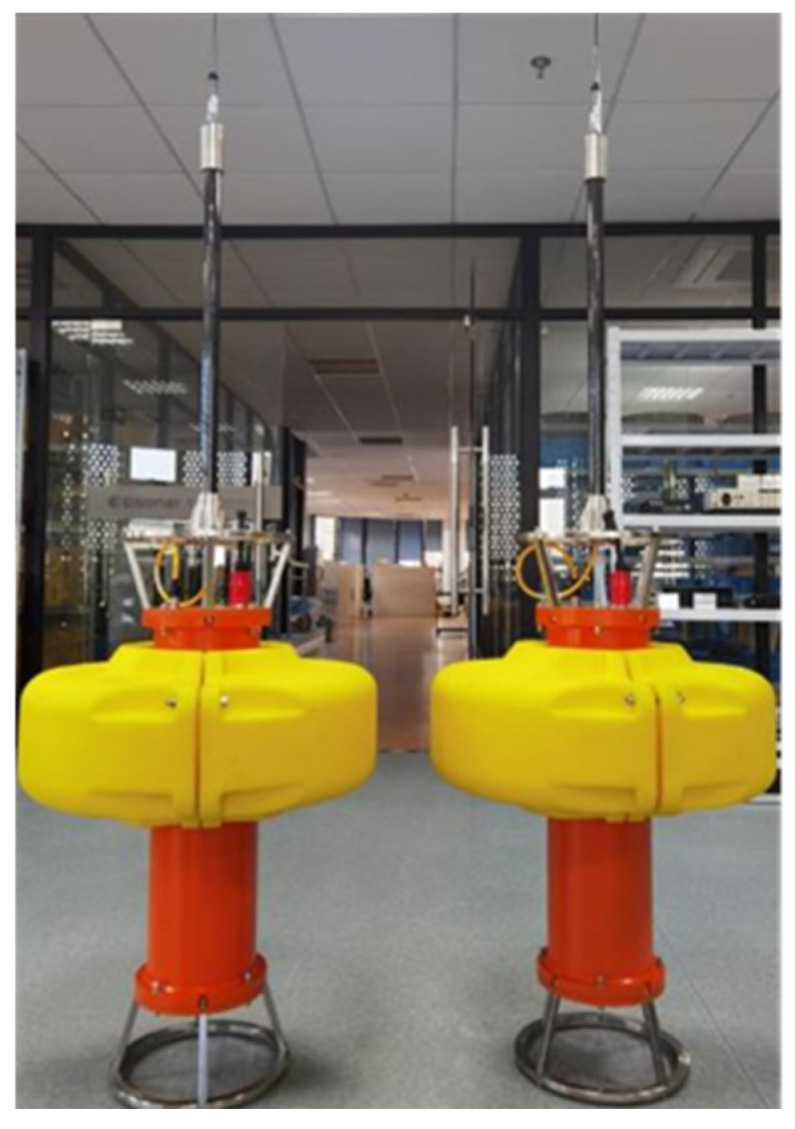
Physical display of data transmission radio communication buoys.

In this design, the main cabin of the buoy is made of aluminum alloy, internally integrating wireless communication modules, power modules, etc. The external structure is equipped with lifting points, and the upper flange can be used to install structures such as antenna support rods. It can be connected to the detection system via cables to ensure the integrity and accuracy of the data. Figure 8 shows the physical display of data transmission radio communication buoys.

**Figure 9:**
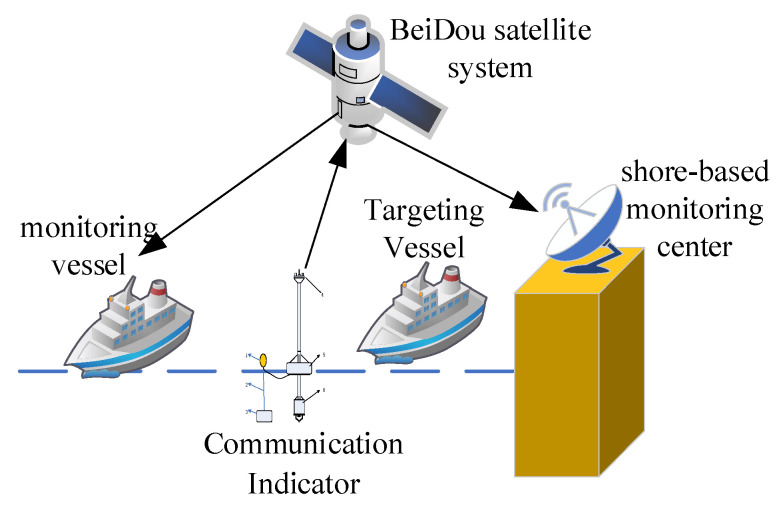
Schematic diagram of data transmission via buoy and Beidou systems.

The data transmission path among the data radio communication buoy, BeiDou satellites, maritime monitoring vessels, and onshore monitoring centers is shown in Figure 9. Leveraging the BeiDou System’s messaging communication function, the BeiDou transmitter terminal within the buoy’s electronic compartment first transmits the ship’s characteristic information to the BeiDou satellite system. The satellite system then relays this information to maritime monitoring vessels and onshore monitoring centers equipped with corresponding BeiDou receiver terminals, thereby fulfilling the monitoring mission of ships passing through a specific sea area [32].

### 2.5. Host Computer Data Processing Module

The seismic signals from the target vessel during navigation contain rich vessel-related information. However, strong ambient seismic noise exists in the target signal frequency band, so noise reduction is needed to extract the target signal for recognition [33].

#### 2.5.1. Adaptive Spectral Line Enhancer

In seismic signal processing, line-spectrum detection is often conducted [34]. The conventional approach of applying the fast Fourier transform to signals yields poor line-spectrum extraction results under low signal-to-noise ratios. Given that seismic signals from ships have low frequencies and are subject to strong broadband background noise, line-spectrum detection is particularly challenging [35]. Therefore, adaptive line-spectrum enhancers are commonly used to boost detection capabilities.

**Figure 10:**
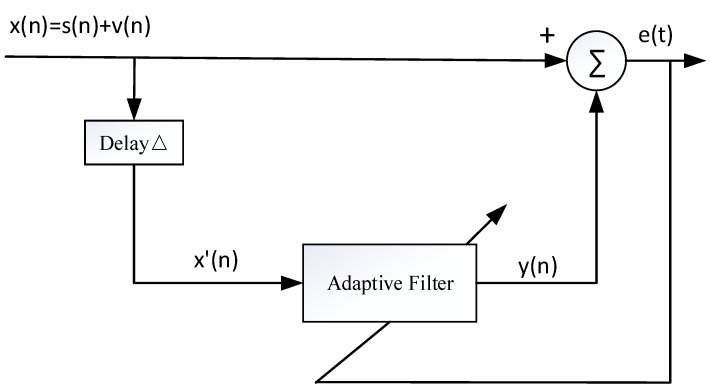
Block diagram of adaptive spectral line enhancement.

An adaptive spectral line enhancer typically consists of two functional parts, including signal separation and spectral line enhancement, as illustrated in Figure 10. The input signal *x(n)* comprises the broadband noise *v(n)* and narrowband signal *s(n),* which are subjected to a certain delay △. The reference signal *x’(n)* is fed into the adaptive filter, yielding an output *y(n)* and error signal *e(n)*. The filter’s weight coefficient vector *w(n)* is adjusted to minimize *e(n)*.

#### 2.5.2. Spectrum Analysis and Peek Detection Mathematical Modeling

Given a discrete signal *x(n)* with N sampling points and a sampling frequency, fs, the Discrete Fourier Transform (DFT) is defined as follows [36]:

(1) $$X\left(k\right)=\sum_{n=0}^{N-1}x\left(n\right)e^{-j2\pi kn/N},k=0,1,\ldots ,N-1$$

The power spectral density (PSD) is defined as follows:

(2) $$P\left(k\right)=\frac{1}{N\cdot f_{s}}{\left|X\left(k\right)\right|}^{2}$$

1. Left/right valley localization:

Search to the left for the first local minimum point $k_{L}$ where $P\left(k\right)<P\left(k-1\right)$.

Search to the right for the first local minimum point $k_{R}$ where $P\left(k\right)<P\left(k-1\right)$.

2. Reference baseline determination:

(3) $$V_{ref}=max\left(P\left(k_{L}\right),P\left(k_{R}\right)\right)$$

3. Salience calculation:

(4) $$P\left(k_{p}\right)=P\left(k_{p}\right)-V_{ref}$$

Adaptive Peak Detection Algorithm. Objective: Extract the first M prominent peaks from P(k) (where M = 3 in the code). Algorithm Steps:

1. Peak candidate identification: Traverse all k values, and mark k as a peak candidate if $P\left(k\right)>P\left(k-1\right)$ and $P\left(k\right)>P\left(k+1\right)$.
2. Salience thresholding: Retain only peaks satisfying $P\left(k_{p}\right)\geq \tau$, where $\tau =\alpha \times \max\left(P\left(k\right)\right)$, $\alpha \in \left(0,1\right)$.

#### 2.5.3. Noise Envelope Signal Recognition (DEMON)

**Figure 11:**
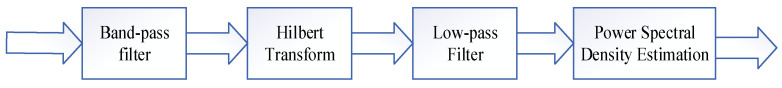
Noise envelope signal recognition analysis flow chart.

Noise envelope signal recognition (DEMON) is a signal-analysis tool based on envelope characteristics [37]. It has shown significant application value in communication, sonar technology, and seismic data analysis [38]. Its core advantage is the effective extraction of useful information and suppression of noise interference. In power spectrum analysis, it extracts line spectra from continuous spectra. In seismic signal analysis, noise can cause signal distortion or overwhelm useful information. DEMON can extract low-frequency signal modulation features from seismic waves. This paper uses the Hilbert transform to extract envelope signals from noise, with the analysis process shown in Figure 11.

1. Original signal: The modulated single-frequency carrier signal is defined as follows:
  (5) $$s\left(t\right)=m\left(t\right)cos\left(\omega_{c}t\right)$$
  where *m*(*t*) represents the low-frequency signal, and *ω_c_* denotes the carrier frequency.
2. Hilbert transform: Perform the Hilbert transform on *s(t)*:
  (6) $$Hs\left(t\right)=m\left(t\right)sin\left(\omega_{c}t\right)$$

The Hilbert transform shifts the phase of each frequency component of the original signal by −90°.

3. Construct the analytic signal by combining the original signal and its Hilbert transform:
  (7) $$z\left(t\right)=s\left(t\right)+jHs\left(t\right)=m\left(t\right)\left[cos\left(\omega_{c}t\right)+jsin\left(\omega_{c}t\right)\right]=m\left(t\right)e^{j\omega_{c}t}$$

The analytic signal contains only positive frequency components, and its spectrum is twice that of the original signal’s positive frequency part.

4. Down conversion for low-frequency signal extraction: Multiply the analytic signal by the complex exponential $e^{-j\omega_{c}t}$:
  (8) $$z_{bb}\left(t\right)=z\left(t\right)e^{-j\omega_{c}t}=m\left(t\right)e^{j\omega_{c}t}\cdot e^{-j\omega_{c}t}=m\left(t\right)$$

This yields the real-valued low-frequency signal *m*(*t*), thus completing the demodulation.

#### 2.5.4. Low-Frequency Analysis (LOFAR)

Low-frequency analysis (LOFAR) obtains a time-varying power spectrum on the time–frequency plane by performing a short-time Fourier transform (STFT) on sampled data [39]. It extracts the line-spectrum distribution characteristics of non-stationary signals from the time–frequency perspective. The process is carried out as follows:

Divide the sampled signal s(n) into K consecutive segments $,s_{j}\left(n\right),$ for j = 1, 2, …, K, with L samples per segment and partial overlap allowed between adjacent segments. Normalize and de-mean each segment’s samples $s_{j}(n)$ as follows:

(9) $$u_{j}\left(n\right)=\frac{s_{j\left(n\right)}}{max\left[s_{j}\left(n\right)\right]}$$

Calculate de-meaning as follows:

(10) $$x_{j}\left(n\right)=u_{j}\left(n\right)-\frac{1}{L}\sum_{i=1}^{L}u_{j}\left(i\right)$$

Calculate the power spectrum for each processed segment:

(11) $$X_{j}\left(k\right)=FFT\left[x_{j}\left(n\right)\right]$$

Plot the power spectra of all segments over time to obtain the LOFAR spectral estimate.

## 3. Experiment and Signal Analysis

According to experimental requirements and on-site environmental conditions, a seismic wave measurement system was constructed using different modules. Prior to testing, relevant preparatory work was carried out to ensure that the system met the experimental requirements. Subsequently, low-frequency sound source reception tests in a water tank and marine field trials were conducted, followed by comprehensive data analysis.

### 3.1. Experiment on Receiving Ultra-Low-Frequency Signal Sources

**Figure 12:**
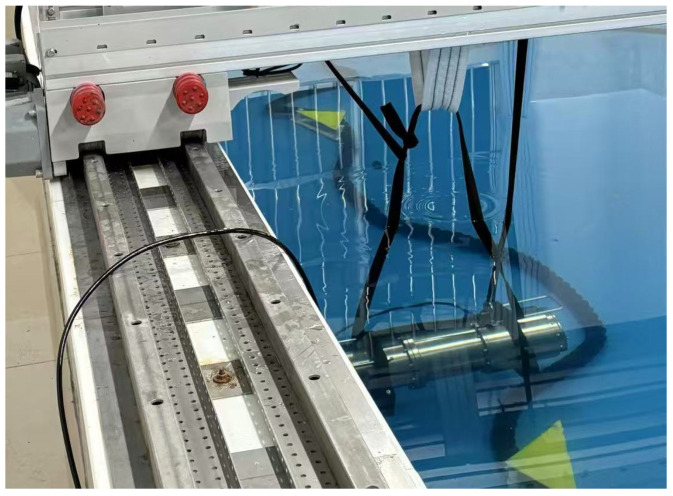
Schematic diagram of low-frequency sound-source lowering.

For the experiment, a low-frequency sound source was configured with an operating frequency range of 10–2000 Hz, spanning the low-frequency (10–20 Hz) and mid-frequency (20 Hz–2 kHz) bands in hydroacoustic classification, which is a sound source level greater than the 150 dB referenced to 1 μPa, and a maximum output power of up to 200 W. Following the technical operation procedures in the debugging phase, the low-frequency sound source was connected by linking the signal generator, power amplifier, and low-frequency sound source via cables. After the connection was made, a thorough inspection was conducted to ensure tight system wiring, particularly at the watertight joints, to prevent water seepage, which could cause short-circuiting and damage to the instruments. Finally, the low-frequency sound source was lowered into the water tank. The lowering process of the low-frequency sound source is shown in Figure 12.

**Figure 13:**
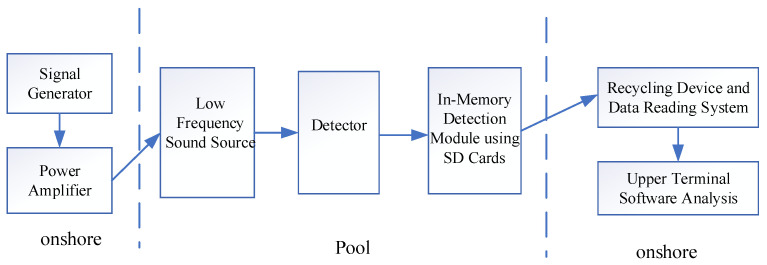
The experimental process in a flow chart.

Due to the shallow depth of the water tank, underwater acoustic communication and buoy devices were not used. Instead, an SD card-based internal recording detection module was employed for the water tank test to verify the perfection of the detection device’s receiving function. The low-frequency sound source signals can be adjusted via a signal generator, amplified by a power amplifier, and then transmitted to the underwater low-frequency sound source through cables. The acoustic signals are converted into seismic wave signals at the water–bottom interface, which are finally received by the seismic wave measurement device and stored on an SD card. After the experiment, the data can be extracted and analyzed using host computer software. The experimental flow diagram is shown in Figure 13.

After preparations, the seismic wave measuring device is lowered to the opposite side of the sound source on the pool bottom. It is placed flat to ensure full contact with the bottom surface for better signal reception.

**Figure 14:**
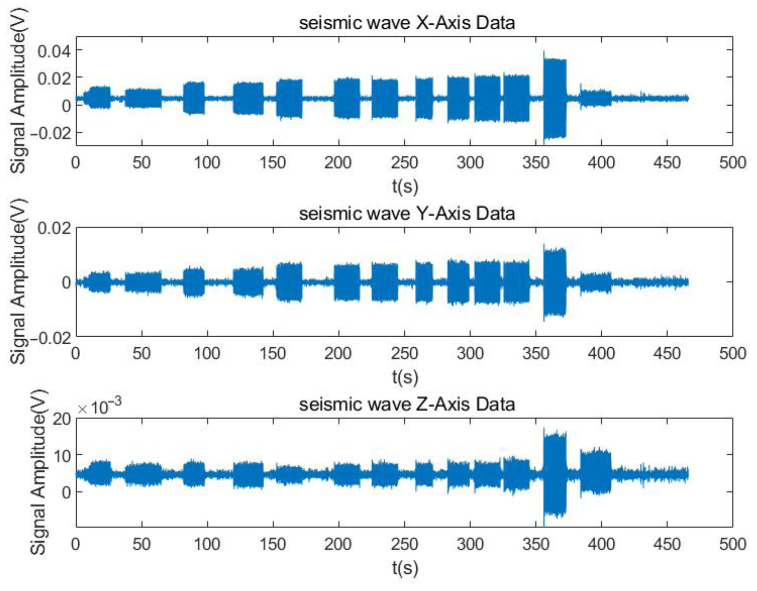
Seismic wave signals from pool tests.

The signal source is set to emit signals, as shown in Figure 14. It sends a 10 s low-frequency acoustic signal every 10–30 s, covering frequencies from 15 Hz to 60 Hz.

After data extraction and plotting a 500 s seismic wave signal is obtained, as shown below.

**Figure 15:**
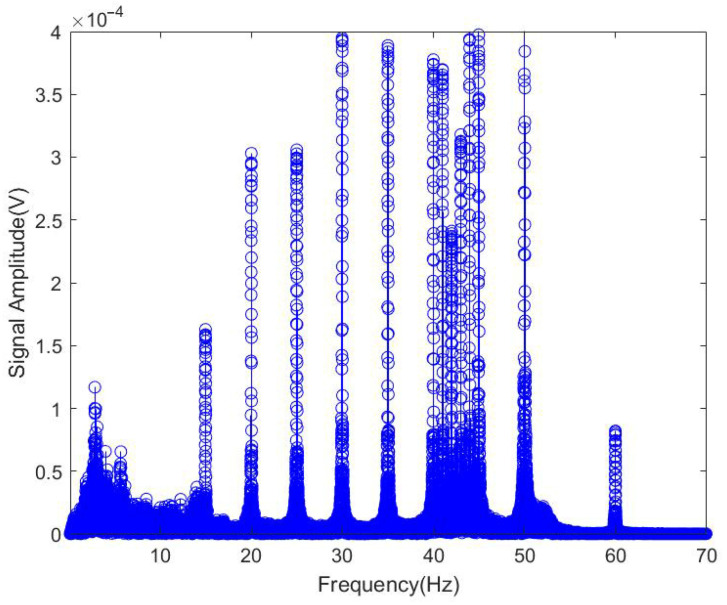
Frequency-domain characteristics of the *x*-axis component.

As can be seen from Figure 15, the seismic detection device correctly receives the number of signals. The Fourier transform of the *x*-axis signal yields the frequency-domain characteristics of the *x*-axis component.

**Figure 16:**
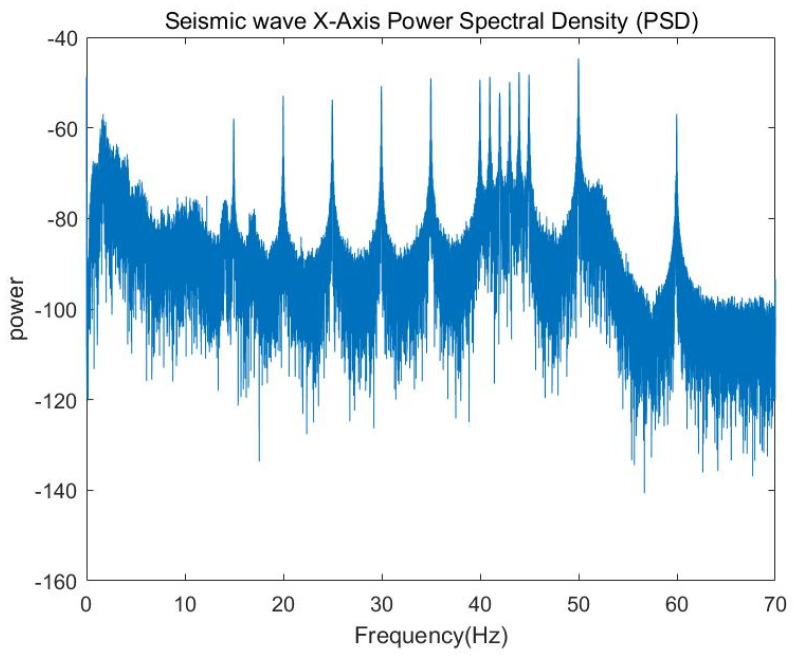
Seismic wave *x*-axis power spectral density.

As can be seen from Figure 15 and Figure 16, the signal received by the seismic wave sensor is basically the same as the emission frequency of the low-frequency sound source.

**Figure 17:**
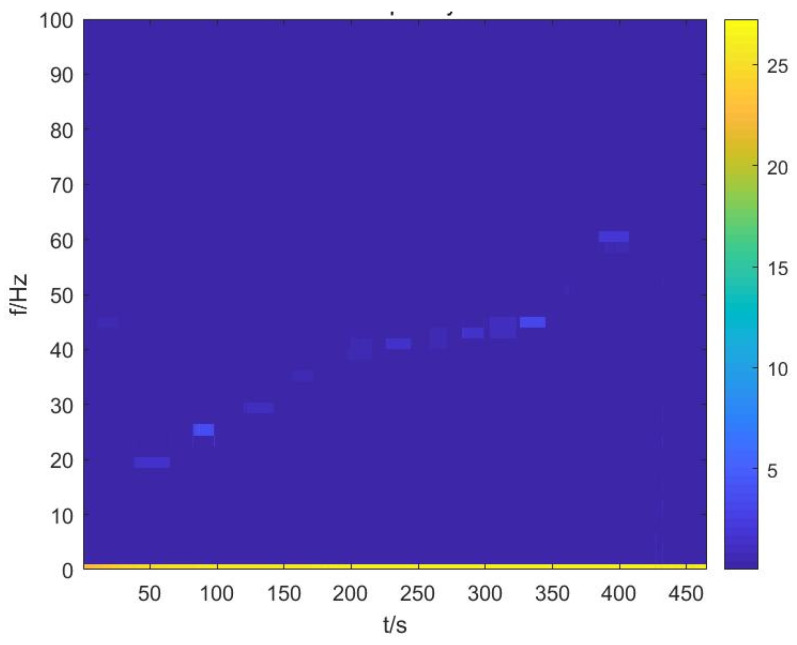
Time–frequency plot of seismic wave signals from water tank test.

A joint time–frequency analysis is then conducted on the seismic waves and the time–frequency features of the very-low-frequency sound source are obtained (see Figure 17).

The obtained frequency domain signal is basically consistent with the time and frequency issued by the low-frequency sound source, which proves that the detection function of the device is normal and fulfills the requirements of the sea trial.

### 3.2. Sea Trials and Host Computer Data Analysis

#### 3.2.1. Experimental Methods and Data Acquisition

**Figure 18:**
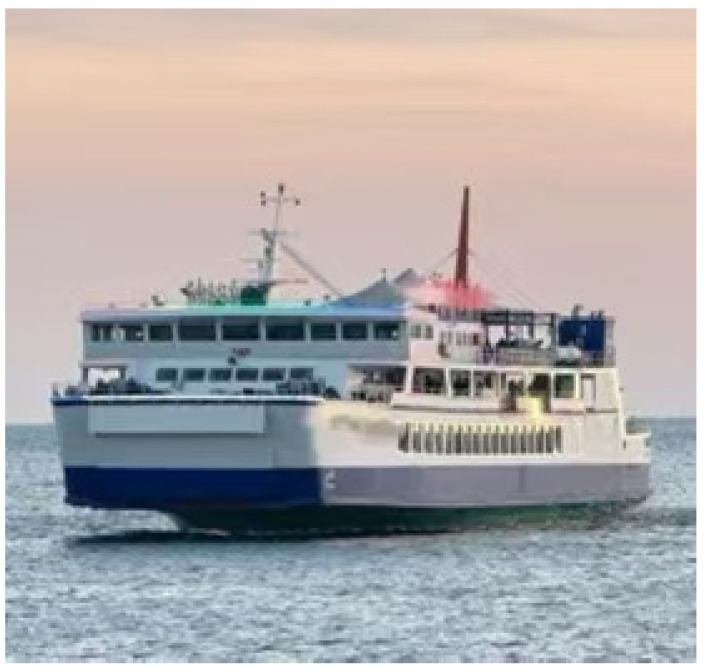
The “Blue Sea Pearl” cruise ship.

After debugging and connecting to the shore, we carried out prefabricated parameters, setting the sampling rate to 1000 Hz, and timed the device through GPS consistent with Beijing time. The device is connected to the buoy so that the upper computer receives the data and then enters the water. The test area was selected as an offshore water area with a water depth of 15 m and a sediment type dominated by sandy clay. The test utilizes an independently designed deep-sea seismic wave monitoring system. Figure 18 shows a cruise ship passing through the sea.

#### 3.2.2. Sea Trial Data Recovery

The housing for the underwater instrumentation module is anchored to the seafloor. An acoustic release ensures vertical stability. The surface buoy receives and relays data to the monitoring center in real time. The system is deployed using a coordinated surface–underwater operation mode:

1. Instrument deployment: Anchor the underwater instrumentation module’s housing to the seafloor. An acoustic release ensures vertical stability.
2. Time synchronization: Use the Beidou satellite timing module (accuracy of ±1 μs) to synchronize the time between the acquisition node and the buoy.

**Figure 19:**
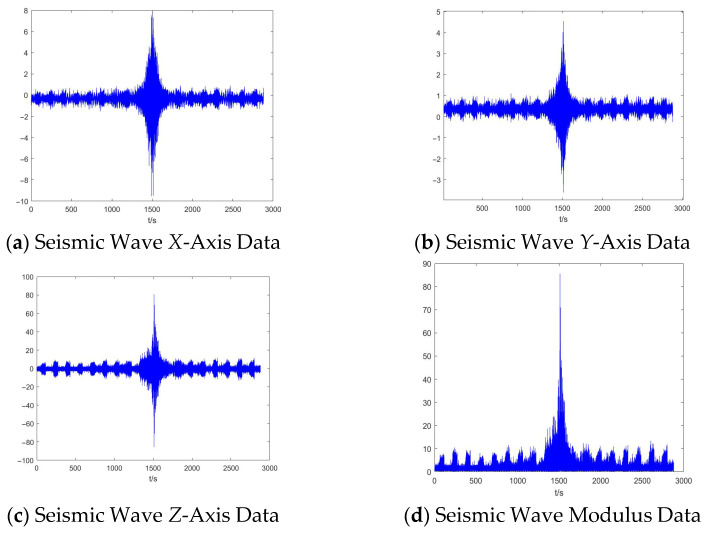
Passage characteristic curve of a ship.

Figure 19 shows the measured characteristic curve of a ship passing through the characteristic curve; after 20 min of deployment, it is clear that there is an obvious ship seismic wave signal passing through the characteristic curve, and the figure shows the time-domain characteristics of the ship on the *x*-axis, *y*-axis, and *z*-axis. The peak value of the signal on the *x*-axis when the target is passing through the measurement point positively and horizontally reaches 7.79 mV; the peak value of the signal on the *y*-axis is about 4.5 mV; and the peak value of the signal on the *z*-axis reaches 80.79 mv The intensity in the vertical direction is significantly greater than that in the horizontal direction.

**Figure 20:**
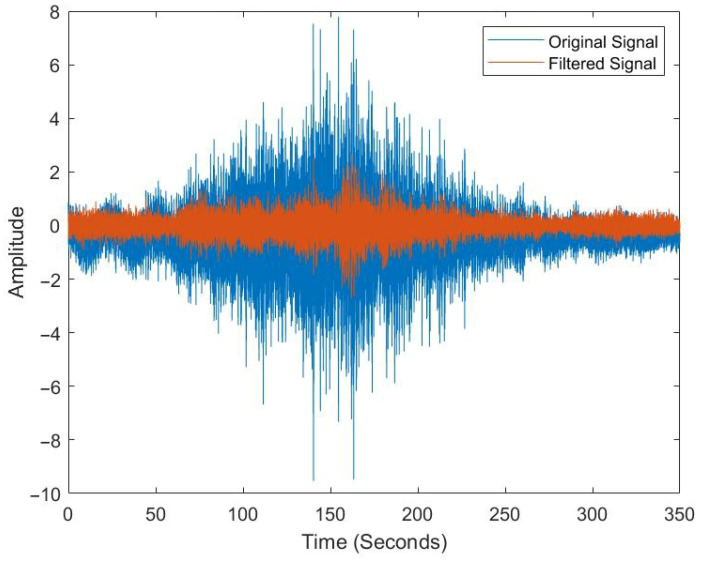
Seismic wave signals after band-pass filtering.

From Figure 16, it can be seen that around 1350–1700 s, there is an obvious presence of a ship through the characteristic curve; in order to facilitate the subsequent signal analysis, the *x*-axis data for 1350–1700 s and the signal range of 1 Hz to 60 Hz are taken for bandpass filtering, and the results obtained are shown in Figure 20:

Conducting a joint time–frequency analysis of the seismic waves obtained and performing a LOFAR spectrum analysis reveal the time–frequency features of the very-low-frequency sound source. However, due to high-level ambient noise, it is challenging to identify seismic wave signals.

**Figure 21:**
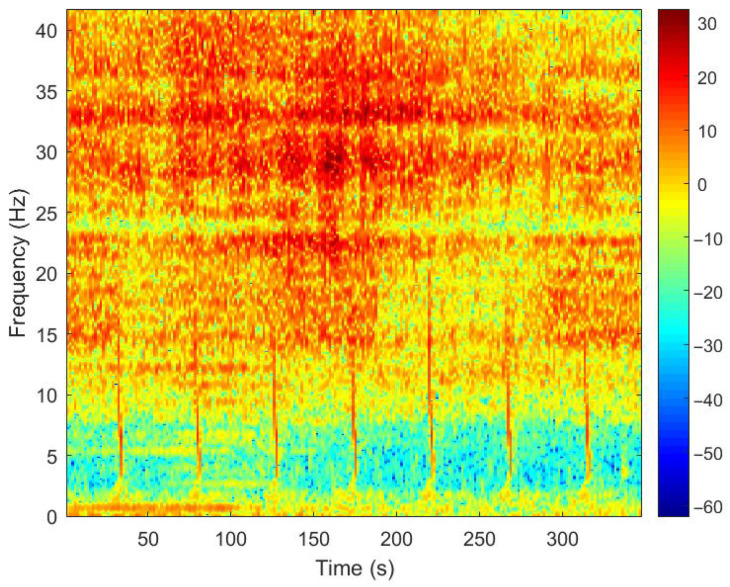
The directly measured time–frequency plot.

**Figure 22:**
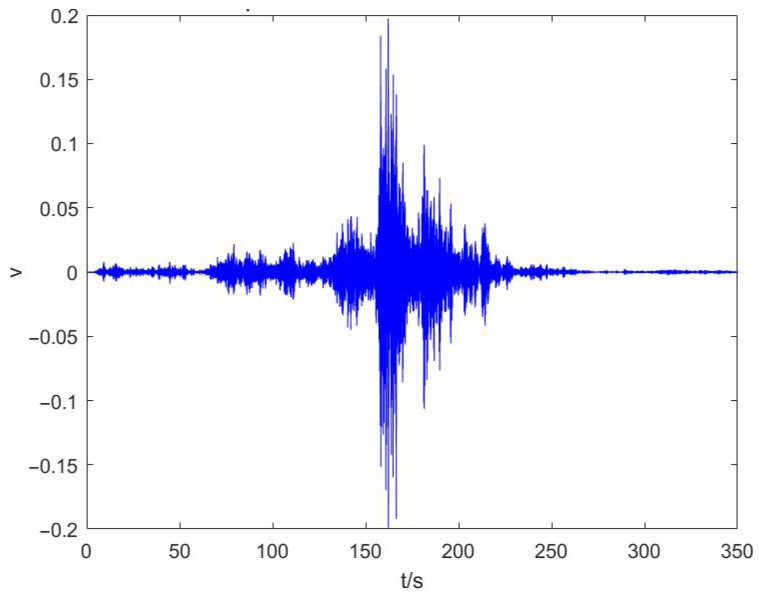
Adaptive seismic wave time-domain plot.

Figure 21 shows the LOFAR spectrum of the unprocessed seismic wave signal. To obtain the seismic wave signal, band-pass filtering and noise reduction are applied. Also, the signal is processed to remove the DC component and detrend it, eliminating low-frequency noise and linear drift. Then, an adaptive spectral line enhancer is employed to extract the spectral lines from the signal. This method leverages the different correlations of radii of the signal and noise. Using an adaptive filter, it extracts the periodic signal from the bandwidth, enhancing the spectral lines. The seismic waveform is obtained, as shown in Figure 22.

**Figure 23:**
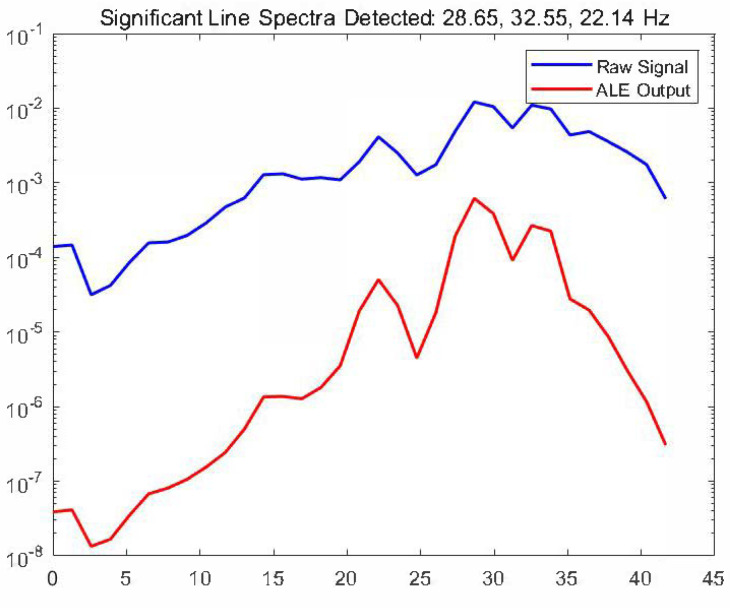
Automatic detection: the significant line-spectrum frequencies detected.

The comparison shows that after ALE processing, the signal’s spectral lines are more distinct, and the background noise is effectively reduced. Subsequent line-spectrum detection reveals noticeable low-frequency spectral lines in the target signal. (The results are shown in Figure 23). Automatic detection identifies significant line-spectrum frequencies at 28.65 Hz, 32.55 Hz, and 22.14 Hz. After processing with the ALE (order 50; step-size factor 0.01), the signal-to-noise ratio in the target band (20–40 Hz) is improved by 12.6 dB.

**Figure 24:**
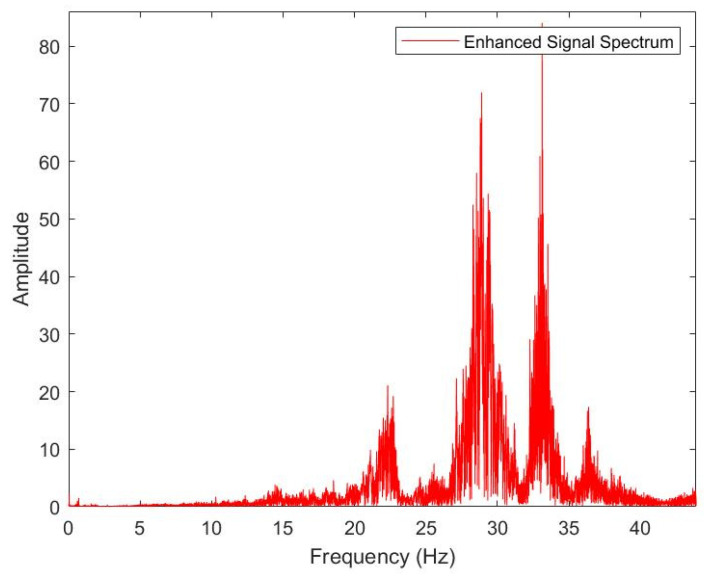
Frequency-domain characteristic curve.

The frequency range of the ship’s seismic wave signals is mainly concentrated in the low-frequency band within the typical seismic signal range. (The results are shown in Figure 24). This confirms that the detection system acquires authentic target seismic wave signals with frequencies below 50 Hz.

**Figure 25:**
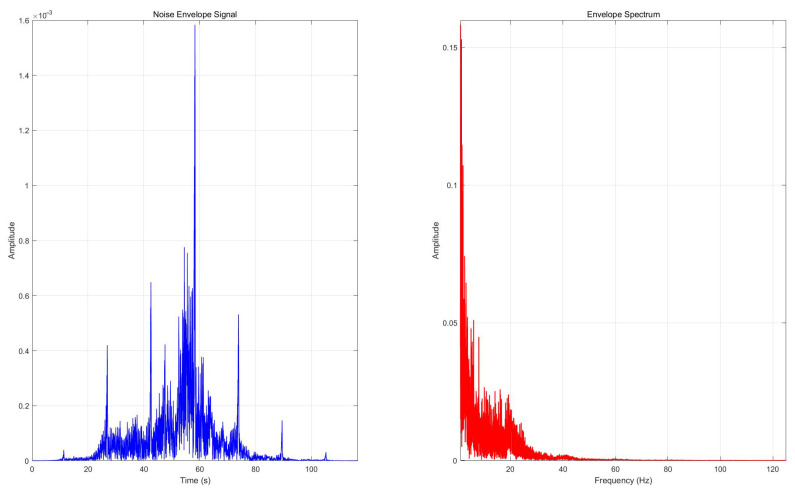
Envelope curve and envelope spectrum curve.

The envelope and envelope spectrum curves of the ship’s signal were obtained. (The results are shown in Figure 25). An analysis of the envelope spectrum shows that it can capture low-frequency noise components in the seismic signal.

The ship’s main envelope spectrum frequencies are 1.99 Hz, 6.02 Hz, and 19.29 Hz, indicating harmonic characteristics. Demodulation reveals submerged low-frequency signals. The ship’s seismic wave fundamental frequency is usually around 2 Hz. This is mainly due to the vibration fundamental frequency and harmonics of the hull mechanical components or the harmonics caused by the propeller’s shaft frequency blade modulation.

**Figure 26:**
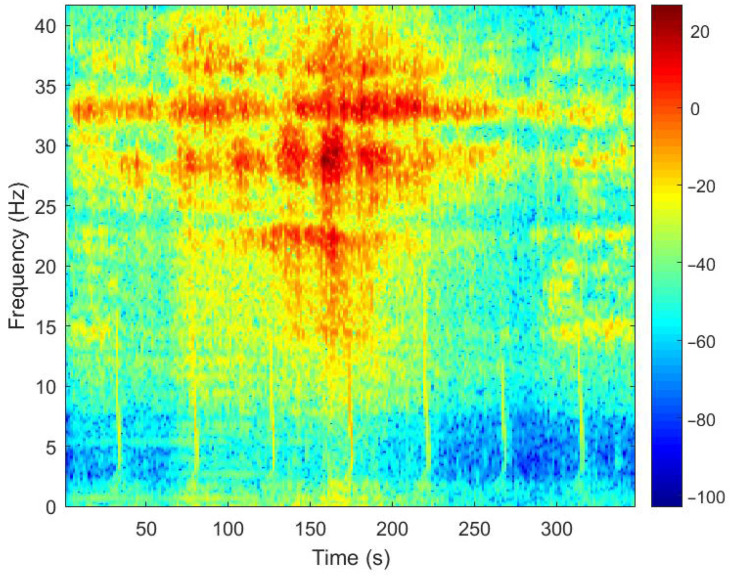
Denoised LOFAR spectrogram.

Figure 26 shows the LOFAR spectrogram of the target signal, showing the distinctive features of the shipboard seismic wave signal at around 20 Hz and 30 Hz. Especially around 50 s, the signal intensity at these frequencies increases markedly. This may indicate that during this period, some ship equipment (such as generators and motors) produced strong vibrations or noise. The LOFAR spectrogram of the target signal shows that the ship-borne seismic wave signals received had strong low-frequency discrete spectral lines, which are clear, numerous, and densely clustered. This is consistent with the characteristics of ship-borne seismic waves.

## 4. Conclusions

This study successfully designed and implemented an underwater monitoring system based on low-frequency seismic wave signals to meet the needs of marine vessel monitoring. This system effectively addresses the key challenges of ship target detection and data transmission through modular architecture and adaptive signal processing technology. The main conclusions are as follows:

The system adopts a surface–underwater collaborative architecture composed of geophones, data acquisition modules, cables, underwater acoustic communication devices, and data transmission radio communication buoys, which facilitates deployment in different sea areas and the recovery of the devices. It constructs an “underwater–surface–remote” large-capacity real-time data transmission network to ensure data integrity and achieve low-cost deployment in shallow sea scenarios.

In signal processing, an adaptive line enhancer is introduced to suppress broadband noise and identify noise envelope signals. By integrating low-frequency spectral analysis to extract time–frequency features, the system significantly enhances line spectrum detection capabilities in low signal-to-noise ratio (SNR) environments. Water tank tests demonstrate that the reception error for 15–60 Hz is less than 2%. Marine field tests successfully captured ship seismic wave signals and achieved remote data transmission via buoy radios.

The ship seismic wavefield exhibits distinct low-frequency line spectrum characteristics and time–frequency properties, which can serve as a critical basis for subsequent underwater detection and early warning applications.

However, the current system has the problem of a limited battery life, and in-depth research will be carried out on this battery life in the future. The next step is to implement the engineering landing of the detection algorithm at the hardware level on the basis of optimizing the battery life and eliminating redundant data collection links through the streamlined design of the data observation process. This technical route will lay a key foundation for the research and development of the subsequent early warning detection system for seismic wavefield signals.

## Figures and Tables

**Table 1:** Detector parameters and performance.

Parameters	Performance	Unit
Sensor Type	High-performance geophone	
Axes	Upward/northward/eastward	
Sensitivity	250	V/m/s
Clipping Level	30	mm/s
Bandwidth	1–300	Hz
Dynamic Range at 1 Hz	110	dB
Cross-Coupling	<1%	
Non-Linearity at 10 Hz	<0.5% of the maximum signal level	
Temperature Range	40 ± 55	°C
Cold Start Time	About 2 min, depending on the bandwidth	min
Maximum Installation Tilt	Any angle	°
Spirit Leveling/Mass Locking	Not required	
Inherent Noise	100	nm/s

## Data Availability

The data presented in this study are available on request from the corresponding author.
